# Global Health Governance and Health Equity in the Context of COVID-19: A Scoping Review

**DOI:** 10.3390/healthcare10030540

**Published:** 2022-03-15

**Authors:** Wafa Abu El Kheir-Mataria, Hassan El-Fawal, Shahjahan Bhuiyan, Sungsoo Chun

**Affiliations:** 1 Institute of Global Health and Human Ecology, The American University in Cairo, New Cairo 11835, Egypt; wafamataria@aucegypt.edu (W.A.E.K.-M.); hassan.elfawal@aucegypt.edu (H.E.-F.); 2 Department of Public Policy and Administration, School of Global Affairs and Public Policy, The American University in Cairo, New Cairo 11835, Egypt; sbhuiyan@aucegypt.edu

**Keywords:** Global Health Governance, equity, COVID-19

## Abstract

Background: Health equity is an important aspect of responsible governance. COVID-19 exposed existing shortfalls of Global Health Governance (GHG). A considerable amount of related literature is produced. This scoping review aims at mapping the present knowledge and at identifying research gaps. Methods: This scoping review is based on the Joanna Briggs Institute’s guideline for standardized methods and PRISMA-ScR guidelines for reporting. Documents published from December 2019 to October 2021 were searched using PubMed, Scopus, Google Scholar, World cat, and WHO-Global Index Medicus. Two reviewers screened and reviewed eligible studies in three stages: duplicates identification and elimination, title and abstract screening, and full-text assessment. Data was charted and results were classified into conceptual categories. Analysis was done in three stages: open descriptive coding, focused thematic analysis, and frequency, commonality and significance analysis. Results: forty-nine studies met the inclusion criteria. Areas of research were grouped into seven themes: “human rights and inequities”, “solidarity, collaboration and partnership”, “GHG structure change”, “political and economic power and finance”, “approaches to address inequity”, “law and regulations”, and “private investment and public-private partnerships (PPPs) in GHG”. The highest number of papers were in the first theme, “human rights and inequities”. However, the themes are interrelated. Authors who contributed to research were mostly affiliated to developed countries indicating a gap in knowledge and expertise in developing countries. Conclusion: Through this scoping review we found that the seven themes are interconnected. Disciplinary collaboration in research relating GHG to health inequities is solicited. Collaboration in research, information sharing, and research capacity development are in needed in developing countries.

## 1. Introduction

Global Health Governance (GHG) is defined as “the use of formal and informal institutions, rules, and processes by states, intergovernmental organizations, and non-state actors to deal with challenges to health that require cross-border collective action to address effectively” [1]. According to Frenk and Moon, GHG has four main functions: First, the production of global goods, guidelines, polices, research and technologies. Second, the management of external threats. Third, the facilitation of global solidarity. Forth, a stewardship function [2]. Health equity is defined as “the absence of unfair, avoidable, or remediable differences among groups of people, whether those groups are defined socially, economically, demographically, geographically, or by other stratifiers (e.g., sex, gender, ethnicity, disability, or sexual orientation)” [3]. GHG has both a moral and a functional role in achieving health equity [4] so that every human being enjoys his right to health. Considering the above GHG functions, health equity could be addressed through each and every one of them. Health equity can be enhanced through better GHG stewardship, better GHG performance in managing of external threats, stronger global solidarity, and more inclusive guidelines and policies. 

Based on the fact that health equity is a moral obligation and is an important aspect of responsible governance, the COVID-19 pandemic has exposed existing shortfalls of GHG through the exacerbation of already existing health inequities across countries as well as within the same country [5]. Since the start of the pandemic, a considerable amount of literature has been produced discussing COVID-19 and health inequities. Different areas have been explored. Some authors discussed inequities between countries and nations, concentrating on the difference between Low and Middle Income Countries (LMICs). LMICs are disproportionality affected by COVID-19 due to their limited capacities and resources and due to High Income Countries (HICs) actions [5]. Other authors concentrated on social determinants of health as reference causes of health inequities [6]. While several authors discussed GHG in relation to health inequities apparent during the COVID-19 pandemic [7,8,9].

Given the ongoing health inequities during the COVID-19 crisis and the experts’ criticism of the current GHG for not ensuring health equity, gathering the knowledge covering these areas and demonstrating the gaps in this knowledge is of high importance to stimulate further research to better understand the status quo and for better future actions. This scoping review aims at mapping the present knowledge and at identifying these gaps. The paper is organized as follows: methodology, detailing the objective and research questions, studies identification, eligibility criteria, and data charting, followed by the results section where results are presented using tables and analyzed thematically. Afterwards, there is a discussion part and finally the conclusion. 

## 2. Materials and Methods

The methodology used in this scoping review is based upon: first, the guidelines for conducting systematic scoping review developed by the Joanna Briggs Institute [10], and second, the Preferred Reporting Items for Systematic Reviews and Meta-Analysis Extension for Scoping Review (PRISMA-ScR). PRISMA-ScR contains 20 essential reporting items and 2 optional items to include when completing a scoping review [11]. These guidelines were used to ensure consistency of results and to enhance utility of the synthesized knowledge. The study was performed in the period between the first of September 2021 up until 25 December 2021.

### 2.1. Objectives and Research Questions

The main objective of the scoping review is to map the body of literature on health equity in relation to GHG in the context of COVID-19. Along with this objective, the scoping review aims at identifying research gaps according to research themes, disciplines, and countries of origin (i.e., authors’ affiliations: developed vs. developing countries). 

### 2.2. Relevant Studies Identification

Relevant studies were identified through searching electronic databases of both published literature and gray literature. Databases used for the search were: PubMed, Scopus, Google Scholar, World cat, and WHO-Global Index Medicus. Key words used for the search were: health equity, health inequity, COVID-19, and global governance. In order to obtain focused results, Boolean operators were used as conjunctions to combine these keywords in the search. The used research term is: “health inequity” OR “health equity” AND “Global Governance” AND “COVID 19”. Keywords were searched in the title, abstract, or keyword.

### 2.3. Eligibility of Studies

Identified studies to be included were to conform with the following inclusion criteria:

- Written in English
- Published starting with the COVID-19 in 2019 up till October 2021
- The main focus is on global governance aspects that affect health equity
- Reports on health equity issue in the COVID-19 context

As for the type of the documents to be included, it has not been limited to peer reviewed article for two reasons: First, the topic (COVID-19) is recent, so not limiting the type of documents included widened the base of search to ensure the collection of as much scholars viewpoints as possible, including the ones that were not developed to full research papers. The second reason is that it is recommended to include gray literature and reports while mapping the research because they are considered valuable sources of information.

Identified eligible studies were screened and reviewed by two reviewers. Screening was done through three stages: Stage one, identified studies were screened to ensure the elimination of any duplicates. Identified studies were imported into Zotero, a citations managing software that can identify duplicates and eliminate them. Stage two: title and abstract screening to exclude ineligible studies (i.e., studies that has the keywords but do not conform with other eligibility criteria). Stage three: full text article assessment of remaining studies in order to confirm their eligibility and include them see Supplementary Materials.

### 2.4. Charting of Data

Data were charted in an Excel table. In the table, the extracted results were classified into conceptual categories according to the objectives of the scoping review. The classification criteria were: year of publication, country, field or discipline, type of publication, aim of the study, relevant findings, and main theme.

### 2.5. Analysis and Results Reporting

Thematic analysis of the data was performed so as to map the present literature. Analysis was done on three stages: First, open descriptive coding to generate ideas. These ideas were to be used in the focused thematic analysis. Second, focused thematic analysis to identify patterns and relationships. Third, frequency, commonality, and significance analysis of previously identified categories. Data distribution is presented using tables along with a narrative descriptive format. 

## 3. Results

**Figure 1:**
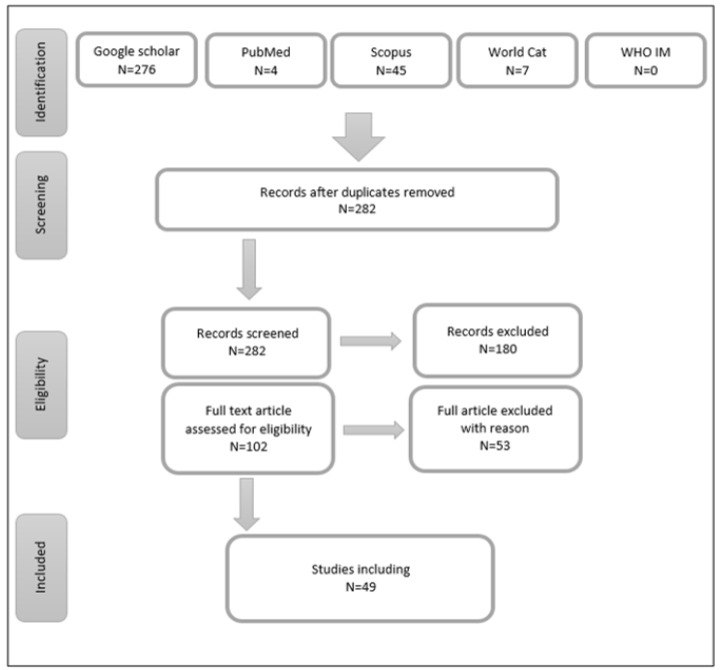
Search flow chart.

The first stage of chosen databases’ search resulted in 332 studies. Following examining for duplicates, 50 studies were eliminated and 282 studies remained. The 282 studies were screened against the inclusion criteria. The primary screening of the title, abstract, and keywords of these articles lead to eliminating 180 studies due to the absence of one or more of the keywords, leaving 102 studies. Within the 102 studies, eight of the sources were not accessible, which led to their exclusion. The rest of the studies, a full text screening was applied and 49 studies met the inclusion criteria (Figure 1).

The characteristics of the included studies are presented in Table 1. 

### Identified Themes

The 49 studies included in this scoping review were found to cover equity in the context of COVID-19 and in relation to GHG in an array of 35 different yet related topic areas. Upon analyzing the different topics, the studies were grouped into seven main proposed themes according to the interconnected areas they cover. The seven themes are: “human rights and inequities”, “solidarity, collaboration and partnership”, “GHG structure change”, “political and economic power and finance”, “approaches to address inequity”, “law and regulations”, and “private investment and PPPs in GHG” (Table 2).

The human rights and inequities theme (theme 1) is the most frequent theme among the included studies. Eleven papers were categorized as part of this theme. The studies highlight the fact that equity is embedded in the essence of human rights in general and in the right to health more specifically. The fact that -the responses to COVID-19 were inequitable- was referred to the values underlying the responses. There proved to be an under-reliance on the human rights as a base in constructing these responses [12,13,14,15]. COVID-19 vaccines are an obvious example of this conduct. Distribution of the vaccine proved to be inequitable [16] as well as other stages of the vaccine production, procurement, and distribution [17]. Not centralizing the COVID-19 responses around human rights could be related to the failure to operationalize human rights in institutions and mode of action of the numerous actors in GHG [18]. Gender mainstreaming in institutions working in the field of GHG especially in emergency response, is required so as to promote equity both at the decision making level as well as at the outcome level [19,20]. Different views and actions on techniques to address equity on the bases of human rights were presented in the studies. COVID-19 Vaccines Global Access (COVAX) as a global initiative is regarded as “the only global solution” to vaccine inequity [21], nevertheless other suggestions emerged such as using digital and medical technology to enhance equity [22]. 

The “structure of GHG and the need for change” theme (theme 3) comprises nine studies out of the 49 studies included. Different authors indicated that the current GHG structure is no longer adequate for facing the present challenges associated with global health and their consequences. Some scholars concentrated on the structural factors that lead to the current health inequities and proposed pro-equity laws and policies [23]. Others indicated that COVID-19 is a turning point that should be used to perform fundamental changes in GHG structure in order to eliminate any barriers to achieve equity [24]. Changes proposed included supporting centralized authority in GHG and binding state rules [25], stronger role for the WHO [26] while concentrating on its professional independent role as global health authority [27], voicing of those that are disproportionately affected [28], inclusive multilateralism and networking in order to leave no one behind [29], flexible collaborative governance to ensure equitable global distribution [30], and creating governance structures with higher representation of the global south [31]. 

Solidarity, collaboration, and partnership theme has five studies out of the 49 included studies. The authors of these studies discussed the need for global solidarity to mitigate COVID-19 effects and consequences through global partnerships, preparedness, and multi-sectoral governance [32]. Others specified the need for global solidarity and collaboration to facilitate capacity bridging between LMICs and HICs [33,34], while some concentrated on solidarity to assure equitable access to COVID-19 vaccines through global initiatives such as COVAX [35,36].

Political and economic power and finance is the fourth theme with nine studies. The majority of these studies discussed economic and political power role in GHG and equity. The authors described power as an access determinant to the COVID-19 vaccine [37] and emphasized the importance of political will for achieving equity [38]. Decisions are shaped by powerful nations, multilateral organizations, and private sectors [39], while other key-group actors have weak participation in decision making [40]. Moreover, the authors emphasized that power through knowledge monopoly—where new methodologies are developed by the Global North and not shared with the South [41]—makes it harder for weaker actors to influence the GHG in the aim of enhancing equity [42]. This leads to prioritizing the wealthier and concentrating on economic recovery rather than on human health and well-being and on inclusive development [6,43]. Lastly, the authors stated that the main causes of inequity were: the presence of unequal power relations [42], the centrality of powerful donors in GHG [44], and market oriented health norms.

The fifth theme “approaches to address inequity” contains four studies. The first study proposes a new approach to address inequity in COVID-19 and its consequences. The approach is basically a global multi-disciplinary human-centered approach aiming at coordinated research, technology development, and health trade facilitation [45]. The second study discusses the use of public health as a base for approaching the current pandemic. The authors argue that public health could support social movements that concentrate on social determinants of health for radical changes. They also state that public health —as a discipline—provides arguments about conditions and decisions that might jeopardize health [46]. The third study proposes a system approach. This approach recognizes the relation between human health, animal health, and environment. It proposes that future interventions need to take these three areas into consideration through a system thinking including all the involved sectors [47]. Lastly, the mutual collective accountability approach, which entails having a shared global governance with a common goal and measurable indicators [11].

The sixth theme “law and regulations” explores the role of law and regulations in GHG as a way to advance global justice and enhance equity [48,49], especially vaccine accessibility [50]. Laws offer legal instruments such as mechanisms, frameworks, and accountability measures to ensure safety and compliance to public health measures [51] as well as laying the foundation to achieve Universal Health coverage, health equity [52], and ensuring timely sharing of information [53]. Intellectual property rights are another topic discussed in this theme. COVID-19 proved that the current intellectual property rights needs modification to allow faster manufacturing and distribution of the vaccine [54]. Lastly, global Public Private Partnerships (PPPs) is another domain where laws need modification or reform. The current global PPPs are not supported by a legal accountability backbone to guarantee: collaboration, benefit sharing [50], and global action rather than nationalistic ones [55].

The last theme is “private investment and PPPs in GHG”. The studies in this theme tackle the topic of prioritizing public interests over economic interests and financial gains. The authors argue that private investors’ and pharmaceutical companies’ main interest is financial gain, which makes PPPs less desirable in GHG as they might lead to inequities [56,57]. Finding financing instruments for GHG to ensure equity can reduce the effects of the private actors and improves equity. COVAX provides a good example on the matter. It enhances accessibility to the vaccine, while considering pharmaceutical companies’ concerns [58].

## 4. Discussion

The scoping review ended with 49 studies. One would assume that—with COVID-19 being the topic of interest of the moment—an enormous number of research papers would be included in this scoping review. However, only 49 studies were eligible to be included. This result might be attributed to the strict methodology used or to the fact that there is a lack of published studies tackling the three areas (GHG, health equity, and COVID-19) at once.

Considering the studies’ characteristics, it is evident that the amount of research increases with time. Most of the research is published in 2021, which is logical given that COVID-19 was detected in the end of 2019 and time is needed to produce data and research. Therefore, as the COVID-19 pandemic continues to exist and time passes, research will accumulate. Looking at the type of publication, most of the included studies are peer-reviewed articles that concentrate on analyzing different aspects combining the three areas. Nevertheless, the results show that around 20% of the included publications are opinion-based pieces such as commentaries, viewpoint, or perspective, which indicate that authors are in the phase of formulating their ideas; however, these ideas did not reach the stage of fully designed research or that there is lack of data and evidence. Discipline wise, most of the studies pertain to the medical field insinuating that certain disciplines are more productive in the area of GHG, equity, and COVID-19 than others. It also indicates that GHG is still thought of as a medical field and not as a multidisciplinary field. As for authors’ countries affiliations, most of the articles with one author originate from developed countries, while multiple authors’ studies have a fairly higher contribution from authors affiliated to developing countries. This can be attributed to: the higher research capacity in developed countries, the need for collaboration and capacity building in developing countries, or the concentration of developing countries’ researchers on the local level research more than the global level research.

**Figure 2:**
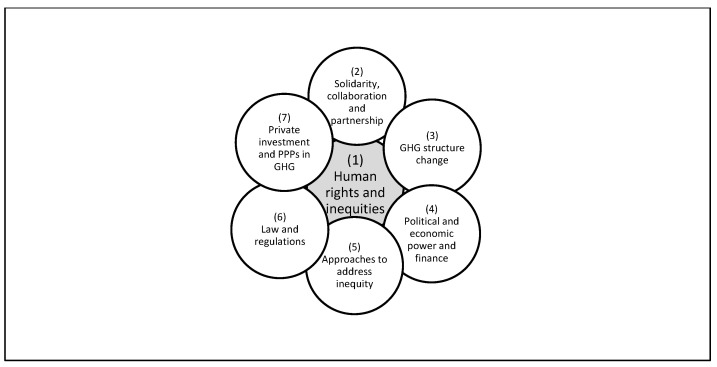
The interlinkages between themes discussing GHG, equity, and COVID-19.

As for the research themes, the authors discuss the values underlying COVID-19 responses, the need for GHG reform, solidarity, and change in GHG power gradient. They deliberate on the role of law and propose new approaches to promote equity. Some also discussed the measures that were taken by the current GHG system to ensure health equity, such as the COVAX initiative. Although each included study has a main theme, many of the articles touch upon other themes showing several points of intersection between the themes. This became apparent following the analysis of the assembled seven themes, it appears that although they concentrate on different domains, they are interrelated. The human rights and inequities theme, the largest theme with 24.49% of the included studies appears to be central to the other themes (Figure 2). Papers discussing GHG structure change in theme 3 debate that the need for structure change emanates from the fact that present inequities are a result from structural factors in GHG that undermine human rights [23] and stressed on the need for building a new GHG structure that promotes equity and human right [29]. In theme 4, the authors touched upon human rights in a different way. They stated that political actors in power need a shift in norms towards human rights and equity so as to produce pro-equity policies [38]. Authors in theme 7 argue that GHG ought to give up the neoliberal values and move towards human rights and equity [56]. Theme 5 calls on multidisciplinary approach founded on the right to health and equity values in order to minimize the protectionism in responses to COVID-19 [45]. In theme 6, the authors urge for a global health law reform to support human rights and equity taking the case of COVID-19 [50]. Finally, theme 2 builds on human rights as a base for global solidarity to ensure equity in response and preparedness to the pandemic [36].

Additionally, several studies across the seven themes seemed to touch on areas in the other themes making the themes interrelated. Articles on solidarity and collaboration (theme 2) called for: multisectorality in GHG structure (theme 3) [32], population-based health initiatives approach to enhance equity (theme 5) [33], overcoming geopolitical power and LMICs in capacity building (theme 4) [34]. Articles on GHG structure change (theme 3) discusses power and hierarchy in decision making (theme 4) [28], collective action (theme 2) [29], and partnership (theme 7) [31]. Articles in theme 7 proposes GHG structure change (theme 3) [56]. Articles in theme 5 relate to theme 2 through discussing research and technology coordination [45].

The interrelation between themes points out that GHG, equity, and COVID-19 are multidimensional and they cannot be limited to one area of analysis, but require a broader thinking approach that builds on different expertise and knowledge from various disciplines.

The themes and the number of articles in each theme is an indication of what the research community is focused on. The focus is on the values underlying the GHG system decisions. Researchers care to emphasize the need to remind the world that the essence of having GHG is to protect humans and their rights, including right to health without any type of discrimination, thus relating GHG to health equity. The authors go further to discuss causes of the inequities, they refer to the present structure of GHG and the power imbalance in this system. They propose several structural changes, higher degree of solidarity and collaboration, and new approaches in GHG to achieve global health equity. However, most of the studies focused on the connection between GHG and health equity through one or two dimensions (i.e., power, authority, law, needed reform, etc.). A holistic approach to equity through GHG should be considered. GHG needs to be considered as a process, starting from inputs to implementation, then outputs, and finally impact. Although health equity comes at the end of the GHG process, health equity needs to be taken into consideration through the whole process: from the beginning of the process, the decision making step (who is participating in the decision making, their power gradient, resources to be employed, presence of regulating laws), to the ways of implementation (production, allocation and procurement), then outcomes (accessibility), and finally the impact (populations health). This holistic approach of streaming equity into the whole process facilitates achieving global solidarity and collaboration to enhance equitable accessibility at the end. Thus, the notion of equity needs to be cultivated in the structure as well as processes of GHG. Only one study mentioned the need to consider the equity aspect at all stages of the vaccine, from production till access.

### Study Limitations

The main limitation of the study is the timeframe. Since COVID-19 is an ongoing pandemic, the research on the topic is still ongoing, which means that more studies will be produced, but not included in this scoping review.

The second limitation is that only English publications were included in the literature search, therefore there is a risk of a language bias.

Lastly, the developed protocol for this scoping review has not been registered.

## 5. Conclusions

The research connecting GHG and health equity in the context of COVID-19 was mapped and grouped into seven main themes: “human rights and inequities”, “solidarity, collaboration and partnership”, “GHG structure change”, “political and economic power and finance”, “approaches to address inequity”, “law and regulations”, and “private investment and PPPs in GHG.” However, it appeared that the themes are interrelated and articles touched on more than one theme. The highest number of papers were in the “human rights and inequities” theme. More research on smaller themes such as the one on solidarity and collaboration is required.

As for research production, the authors contributed to research connecting GHG and health equity in the context of COVID-19 as mostly affiliated to developed countries, indicating a gap in knowledge and expertise in developing countries. This entails the need for information sharing between countries as well as capacity building in developing countries.

Research concerning GHG and equity is multidimensional, which requires a wide range of expertise. There are few multidisciplinary studies in this domain indicating the need for multidisciplinary collaborative research in this area.

## Figures and Tables

**Table 1:** Included studies characteristics.

Study Characteristics *N* = 49				Count (%)	
Year of publication				*N*	%
	2019			3	6.12%
	2020			14	28.57%
	2021			32	65.31%
Type of publication					
	Journal article	Commentary	8	42	85.71%
		Viewpoint	1		
		Perspective	1		
		Analytical	22		
		Essay	3		
		Review	4		
		Systematic review	1		
		Learning module	1		
		Commission report	1		
	Book chapter			3	6.12%
	Background paper			1	2.04%
	PhD Thesis			1	2.04%
	Discussion paper			1	2.04%
	Document			1	2.04%
Discipline					
	Medicine			25	
	Bioethics and humanities			1	
	Social sciences			9	
	Development and policy			1	
	Law and policy			6	
	Communication			1	
	Economics and political sciences			2	
	Multidisciplinary			4	
Country					
	Single country			32	65.31%
	UK			4	
	USA			9	
	Australia			3	
	Taiwan			1	
	Germany			1	
	Italy			1	
	Nigeria			1	
	Canada			5	
	Finland			1	
	India			1	
	China			1	
	Netherlands			1	
	Sri Lanka			1	
	Norway			2	
	Two or more countries			13	26.53%
	France, UK			1	
	Norway, UK			1	
	UK, US, Sweden			1	
	Belgium, India, Guinea, Peru			1	
	UK, Rwanda			1	
	UK, USA, Lithuania, Kenya, Switzerland			1	
	USA, Zimbabwe, Mexico, Belgium			1	
	UK, USA, Kenya			1	
	UK, Australia			1	
	Australia, UK, USA			1	
	Bangladesh, Sweden, Uganda, US			1	
	New Zealand, Hong Kong			1	
	US, South Africa, India, Australia			1	
	Unidentified/commission/UN			4	8.16%

**Table 2:** Main themes.

Main Theme			*N*	%
1	Human rights and inequities		11	22.49%
	Right to health and human rights	3		
	COVAX as a charitable PPP’s model to enhance equity	1		
	Digital technology role in enhancing equity, medical technology	1		
	Decolonizing GHG/right based approach	1		
	Inequity through different stages of vaccine	1		
	VALUES to consider in governing global vaccine distribution	2		
	Gender mainstreaming in IOs, in policy and response	2		
2	Solidarity, collaboration, and partnership		5	10.20%
	Solidarity through COVAX, technology transfer and voluntary license-sharing	1		
	Weak solidarity as a cause for inequity	1		
	GH partnership	1		
	Capacity bridging, collaboration, population-based health initiatives are needed to face inequity	1		
	Improving capacity in LMICs	1		
3	GHG structure change		9	18.37%
	Structural factors for health inequity	1		
	Many actors, no centralized authority, nor binding rules	1		
	Flexible governance, adequate financing, and evidence-based, collaborative	1		
	Justice and equity as the principle for GH practice	1		
	Unequal power relation/move some power to global south	1		
	Power, resources, and networks in GHG policy formulation	1		
	WHO—stronger independent structure to ensure equity	2		
	Inclusive multilateralism	1		
4	Political and economic power and finance		9	18.37%
	Political will and pro-equity policies	2		
	Centrality of power in GHG	1		
	Power and political economy/power as an access determinant to the vaccine	4		
	Quitting one-size-fits-all approach in equity, tends to prioritize the interests of HICs	2		
5	Approaches to address inequity		4	8.16%
	Multi-disciplinary effort is needed	1		
	Public health centrality in decision making	1		
	Global system approach	1		
	Mutual collective accountability	1		
6	Law and regulations		8	16.33%
	Health security and IHR to enhance equity	1		
	Role of law	1		
	Global intellectual property rules modification	1		
	Inequitable information sharing IS/international law for IS	1		
	Law capacity to advance GH justice	1		
	GH law reform	3		
7	Private investment and PPPs in GHG		3	6.12%
	Financial instrument for GHG—private investors renders GHG more secretive	1		
	Less PPP in GHG PPP causes inequity	2		

## Data Availability

Data sharing is not applicable to this article.

## Supplementary Materials

The following supporting information can be downloaded at: https://www.mdpi.com/article/10.3390/healthcare10030540/s1.
