# Supplementary material for: Global Health Governance and Health Equity in the Context of COVID-19: A Scoping Review
Source: Healthcare (Basel). 2022 Mar 15;10(3):540. doi: 10.3390/healthcare10030540 (PMC8949542; doi:10.3390/healthcare10030540)
Supplement: Supplementary file 1 [file healthcare-10-00540-s001.zip › healthcare-1561453-supplementary.pdf]

The list of authors' names, study titles, and year of publication

Title	Author	Year
The right to health must guide responses to COVID-19	Pūras, Dainius; de Mesquita, Judith Bueno; Cabal, Luisa; Maleche, Allan; Meier, Benjamin Mason	2020
Policy opportunities to enhance sharing for pandemic research	Rourke, Michelle; Eccleston-Turner, Mark; Phelan, Alexandra; Gostin, Lawrence	2020
Solidarity and universal preparedness for health after covid-19	Tomson, Göran; Causevic, Sara; Ottersen, Ole Petter; Peterson, Stefan Swartling; Rashid, Sabina; Wanyenze, Rhoda Kitti; Yamin, Alicia Ely	2021
Digital Technology and the Political Determinants of Health Inequities: Special Issue Introduction	Storeng, Katerini Tagmatarchi; Fukuda-Parr, Sakiko; Mahajan, Manjari; Venkatapuram, Sridhar	2021
Covid-19 pandemic and the social determinants of health	Paremoer, Lauren; Nandi, Sulakshana; Serag, Hani; Baum, Fran	2021
Creating political will for action on health equity: practical lessons for public health policy actors	Baum, Fran; Townsend, Belinda; Fisher, Matthew; Browne-Yung, Kathryn; Freeman, Toby; Ziersch, Anna; Harris, Patrick; Friel, Sharon	2020
COVID-19 and the moral imagination	Patel, Mahomed Said; Phillips, Christine Beatrice	2021
Challenges to Global Health Governance from the International Trade in Organ Transplants: Time for a New Model?	Langran, Irene	2021
What lessons does the COVID-19 pandemic hold for global alcohol policy?	Leung, June Yue Yan; Yeung, Shiu Lun Au; Lam, Tai Hing; Casswell, Sally	2021
Human rights and fair access to COVID-19 vaccines: the International AIDS Society–Lancet Commission on Health and Human Rights	Beyrer, Chris; Allotey, Pascale; Amon, Joseph J.; Baral, Stefan D.; Bassett, Mary T.; Deacon, Harriet; Dean, Lorraine T.; Fan, Lilianne; Giacaman, Rita; Gomes, Carolyn	2021
Global health in a turbulence time: a commentary	Lee, Tsung-Ling	2020
Strengthening Global Health Security and Reforming the International Health Regulations: Making the World Safer From Future Pandemics	Blinken, Antony J.; Becerra, Xavier	2021
We need people's WHO to solve vaccine inequity, and we need it now	Kim, Hani	2021
Facilitating Access to a COVID-19 Vaccine through Global Health Law	e ath Law, Global	2019
Advancing human rights through global health governance	Meier, Benjamin Mason; Cinà, Margherita Marianna; Gostin, Lawrence O.	2020

The Breathing Catastrophe: COVID-19 and Global Health Governance	Dentico, Nicoletta	2021
Building back fairer in public health policy requires collective action with and for the most vulnerable in society	D'Ambruoso, Lucia; Abbott, Pamela; Binagwaho, Agnes	2021
Global health in low-income and middle-income countries: a framework for action	Olufadewa, Isaac; Adesina, Miracle; Ayorinde, Toluwase	2021
Equity at a time of pandemic	Plamondon, Katrina M.	2021
COVID-19 vaccine equity: a health systems and policy perspective	van de Pas, Remco; Widdowson, Marc-Alain; Ravinetto, Raffaella; NS, Prashanth; Ochoa, Theresa J.; Fofana, Thierno Oumar; Van Damme, Wim	2021
From Ebola to COVID-19: what explains institutionalized manias and the ultimate preference for non-optimal solutions in global health governance?	Ahen, Frederick	2021
The Case for a Global Healthcare Partnership	Kher, Rajeev; Nair, Arun S.	2020
COVID-19, poverty and inclusive development	Gupta, Joyeeta; Bavinck, Maarten; Ros-Tonen, Mirjam; Asubonteng, Kwabena; Bosch, Hilmer; van Ewijk, Edith; Hordijk, Michaela; Van Leynseele, Yves; Cardozo, Mieke Lopes; Miedema, Esther	2021
In September 2020, the World Health Organization called for ‘equitable access to COVID-19 tools’ which included ‘the development, production and equitable access to COVID-19 tests, treatments and vaccines globally, while strengthening health systems’. What evidence is there of inequity of access so far in this pandemic? What is the role of national governments and of the WHO in this?	Osborne, Rhiannon	2021
Understanding geopolitical determinants of health	Persaud, Albert; Bhugra, Dinesh; Valsraj, Koravangattu; Bhavsar, Vishal	2021
Global Health Partnerships and Translation	Chattu, Vijay Kumar; Aslanyan, Garry	2020
Vaccine ethics: an ethical framework for global distribution of COVID-19 vaccines	Jecker, Nancy S.; Wightman, Aaron G.; Diekema, Douglas S.	2021
Risky business: COVAX and the financialization of global vaccine equity	Stein, Felix	2021
WHO runs the world–(not) girls: gender neglect during global health emergencies	Wenham, Clare; Davies, Sara E.	2021
Governance and COVID-19: a background paper for the 2021 SDG 16 conference	Steven, A.; Williams, M.	2020
The practice of evaluating epidemic response in humanitarian and low-income settings: a systematic review	Warsame, Abdihamid; Murray, Jillian; Gimma, Amy; Checchi, Francesco	2020

We Cannot Win the Access to Medicines Struggle Using the Same Thinking That Causes the Chronic Access Crisis	Krikorian, Gaëlle; Torreele, Els	2021
The legal determinants of health: harnessing the power of law for global health and sustainable development	Gostin, Lawrence O.; Monahan, John T.; Kaldor, Jenny; DeBartolo, Mary; Friedman, Eric A.; Gottschalk, Katie; Kim, Susan C.; Alwan, Ala; Binagwaho, Agnes; Burci, Gian Luca	2019
Imagining Global Health with Justice: Transformative Ideas for Health and Well-Being While Leaving No One Behind	Gostin, Lawrence O.; Friedman, Eric A.	2019
Special Volume No. 1, 2021: The Global One Health Environment	Abed, Yehia; Sahu, Monalisha; Ormea, Veronica; Mans, Linda; Lueddeke, George; Laaser, Ulrich; Hokama, Tomiko; Goletic, Rusmir; Eliakimu, Eliudi; Dobe, Madhumita	2021
Development Trends and Assistance for Health	Zwi, Anthony B.	2020
Ethical, public health, and economic dimensions of the inequitable global distribution of COVID-19 vaccines	Pranav Tandon	2021
Public health and degrowth working synergistically: what leverage for public health	Ouimet, M. O.; Turcotte, P. L.; Rainville, L. C.; Abraham, Y. M.; Kaiser, D.; Badillo-Amberg, I.	2021
COVID-19 Trends Among Persons Aged 0–24 Years — United States, March 1–December 12, 2020<br>A Case Study of the<br>First Year	Sepúlveda, Jaime	2021
The Politics of Medicine: Power, Actors, and Ideas in the Making of Health	Winiarek, Claire Wulf	2021
Fragmented health systems in COVID-19: rectifying the misalignment between global health security and universal health coverage	Lal, Arush; Erondu, Ngozi A.; Heymann, David L.; Gitahi, Githinji; Yates, Robert	2021
COVID-19 reveals weak health systems by design: Why we must re-make global health in this historic moment	Shamasunder, Sriram; Holmes, Seth M.; Goronga, Tinashe; Carrasco, Hector; Katz, Elyse; Frankfurter, Raphael; Keshavjee, Salmaan	2020
Global public health security and justice for vaccines and therapeutics in the COVID-19 pandemic.	Hotez PJ, Batista C, Amor YB, Ergonul O, Figueroa JP, Gilbert S, Gursel M, Hassanain M, Kang G, Kaslow DC, Kim JH, Lall B, Larson H, Naniche D, Sheahan T, Shoham S, Wilder-Smith A, Sow SO, Strub-Wourgaft N, Yadav P, Bottazzi ME	2021
The construction and reconstruction of global health governance system under public health emergencies	Lou, L.; Wei, Y.; Wei, X.	2020

COVID-19 and future pandemics: a global systems approach and relevance to SDGs	Thoradeniya, Tharanga; Jayasinghe, Saroj	2021
The World Health Organization in Global Health Law	Meier, Benjamin Mason; Taylor, Allyn; Eccleston-Turner, Mark; Habibi, Roojin; Sekalala, Sharifah; Gostin, Lawrence O.	2020
Rethinking the central role of equity in the global governance of pandemic response	Eyawo, Oghenowede; Viens, A. M.	2020
COVAX and the rise of the ‘super public private partnership’ for global health	Storeng, K.T., de Bengy Puyvallée, A., Stein, F.	2021
THE URGENCY OF MUTUAL COLLECTIVE ACCOUNTABILITY IN GLOBAL HEALTH GOVERNANCE.	Kohler, J. C.	2021
